# Nymphal RNAi: systemic RNAi mediated gene knockdown in juvenile grasshopper

**DOI:** 10.1186/1472-6750-5-25

**Published:** 2005-10-03

**Authors:** Ying Dong, Markus Friedrich

**Affiliations:** 1 Department of Biological Sciences, Wayne State University, 5047 Gullen Mall, Detroit, MI 48202, USA; 2 Center of Developmental Biology, UT Southwestern Medical Center, 5323 Harry Hines Blvd, Dallas, TX 75390, USA

## Abstract

**Background:**

Grasshopper serves as important model system in neuroscience, development and evolution. Representatives of this primitive insect group are also highly relevant targets of pest control efforts. Unfortunately, the lack of genetics or gene specific molecular manipulation imposes major limitations to the study of grasshopper biology.

**Results:**

We investigated whether juvenile instars of the grasshopper species *Schistocerca americana *are conducive to gene silencing via the systemic RNAi pathway. Injection of dsRNA corresponding to the eye colour gene *vermilion *into first instar nymphs triggered suppression of ommochrome formation in the eye lasting through two instars equivalent to 10–14 days in absolute time. QRT-PCR analysis revealed a two fold decrease of target transcript levels in affected animals. Control injections of EGFP dsRNA did not result in detectable phenotypic changes. RT-PCR and *in situ *hybridization detected ubiquitous expression of the grasshopper homolog of the dsRNA channel protein gene *sid-1 *in embryos, nymphs and adults.

**Conclusion:**

Our results demonstrate that systemic dsRNA application elicits specific and long-term gene silencing in juvenile grasshopper instars. The conservation of systemic RNAi in the grasshopper suggests that this pathway can be exploited for gene specific manipulation of juvenile and adult instars in a wide range of primitive insects.

## Background

Including some of the most persistent and devastating agricultural pests such as the African migratory locust *Schistocerca gregaria*, acridid orthopterans represent a group of insects with significant economic impact and a target of major research efforts [1-3]. Species of the grasshopper genus *Schistocerca *serve also as powerful model system in neurosciences, development and evolution [4-7]. The development of methods facilitating molecular manipulation of these primitive insects is thus of wide interest. However, until now successful approaches for a specific interference with gene expression have not been reported. Here we report results from investigating the conservation of the systemic RNAi pathway in the juvenile (nymphal) form of the grasshopper species *Schistocerca americana*.

RNA interference (RNAi) or the degradation of specific mRNA species in response to cytosolic presentation of sequence identical dsRNA molecules is a widespread phenomenon among eukaryotes. Following its discovery in *C.elegans *[8], RNAi has been widely adopted as powerful loss of function gene analysis tool. Components of the intracellular RNAi pathway machinery like the dsRNA processing enzyme Dicer, and the RNA induced silencing complex (RISC) have been found in many eukaryote model organisms [9]. Less is known yet regarding the conservation of mechanisms facilitating the amplifying and systemic nature of RNAi in *C. elegans*. Systemic RNAi describes the fact that extracellular application of dsRNA via body cavity injection, soaking, or feeding leads to global and persistent gene silencing in treated individuals and their progeny [10,11]. The systemic RNA pathway involves cellular dsRNA uptake and very likely also cellular amplification and release of dsRNA [9].

Recent molecular genetic efforts in *C.elegans *identified the *systemic RNA interference-deficient-1 *(*sid-1*) gene as essential and sufficient for the systemic induction of RNAi [12,13]. *Sid-1 *encodes a seven helix transmembrane protein which has been shown to function as a channel for the uptake of dsRNA molecules and may also facilitate the release of dsRNA from cells [13]. Homologs of *sid-1 *with the capacity to enhance the systemic uptake of dsRNA have been reported from humans but not yet from other organisms [13,14]. However, reports of gene knockdown following systemic application of dsRNA in phylogenetically distantly related animal species such as flatworms, annelids and insects suggests that the systemic RNAi pathway is widely conserved [15-18]. In the red flour beetle *Tribolium *castaneum for example, injection of dsRNA in the body cavity of the parental females (parental RNAi) or final instar larvae (larval RNAi) leads to induction of specific gene silencing in embryos and pupae respectively [17,18]. Efforts to trigger similar gene silencing effects in the fruit fly *Drosophila melanogaster *have failed [19]. Intriguingly, the lack of systemic RNAi competence correlates with absence of a *sid-1 *homolog from the *Drosophila *genome, while *sid-1 *homologous sequences are present in the EST database of the systemic RNAi competent insect species (see below). Experimental and phylogenetic evidence thus identify *sid-1 *as a conserved facilitator of systemic RNAi.

## Results

### Ubiquitous expression of a *sid-1 *dsRNA channel protein gene homolog in grasshopper

Considering the causal relationship between *sid-1 *expression and systemic RNAi competence, we investigated the presence of *sid-1 *in *Schistocerca*. Parts of a *sid-1 *homologous grasshopper gene (*Sa_sid-1*) were isolated by degenerate PCR from nymphal cDNA using a set of nested primer pairs designed against the C-terminal region of *sid-1*, which is strongly conserved between *sid-1 *of *C.elegans *and *sid-1 *homologous sequences identified in EST databases of human and honey bee (Additional File 1). Subsequent RT-PCR experiments with a single, specific primer combination amplified grasshopper *sid-1 *from cDNA of mid-stage embryos, nymphal head or abdomen, and adult eye, leg and ovaries (Additional File 2). Whole mount *in situ *hybridization with a *Sa_sid-1 *probe against mid-stage embryos revealed ubiquitous expression at uniform levels (not shown). In combination, these results demonstrated that grasshopper possesses a homolog of *sid-1*, which is expressed in ubiquitous and uniform manner.

### Systemic RNAi induced knockdown of the grasshopper eye coloration gene *vermilion*

**Figure 1:**
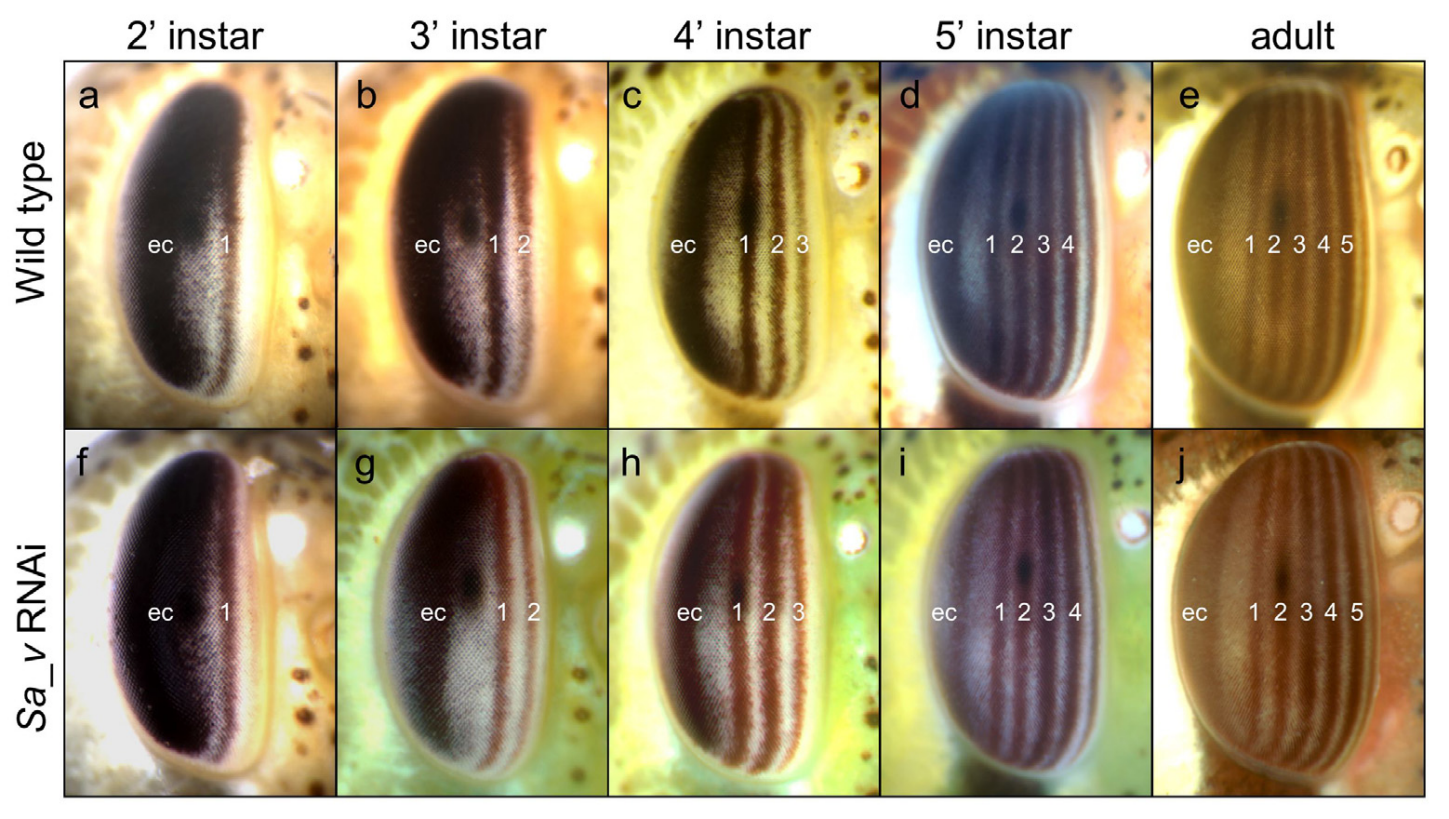
**Time course of eye coloration change in *Sa_v *knockdown grasshopper nymphs. **Anterolateral views of wild type (a-e) and *Sa_v *knockdown (f-j) grasshopper eyes at different stages of development. Anterior is to the right and dorsal up. Numbers denote instar during which the corresponding vertical eye stripe has been formed. (a,f) second instar. (b,g) third instar. (c,h) fourth instar. (d,i) fifth and final nymphal instar. (e,j) adult eye. Note bright red brown coloration of stripes one and two in panels g and h compared to dark brown coloration in the embryonic cap (ec), the pigmented compound eye area formed in the embryo. Also in the adult eye of the *Sa_v *knockdown animal shown in panel j, stripes one and two are more lightly coloured than the later, more anterior stripes.

To be able to test if grasshopper nymphs are conducive to RNAi mediated gene silencing, we cloned part of the *S.americana *ortholog of the *Drosophila *eye coloration gene *vermilion *(*Sa_v*) (Additional File 3). The *v *gene encodes tryptophan oxygenase, which catalyzes the degradation of tryptophan to kynurenine, the primary substrate of the ommochrome pigment synthesis pathway [20]. *v *mutant *Drosophila *are characterized by brighter red coloration in the adult compound eye due to lack of the dark ommochrome pigment and the presence of a second, pteridine based pigment [21]. Grasshopper compound eyes also contain red pteridine and dark ommochrome pigments, which are coexpressed in five vertical stripes (Fig. 1a–e) [22]. Each stripe is formed during one of the five nymphal instars. The number of stripes at a given instar thus corresponds to the number of previously completed instars rendering eye coloration a convenient readout for assaying the temporal dynamics of gene perturbation. *Sa_v *dsRNA was prepared by bidirectional *in vitro *transcription from template DNA prepared by PCR amplification with primers targeting the vector internal transcription start sites. This strategy resulted in 698 bp long dsRNA covering the entire cloned tryptophane oxygenase open reading frame region (see Additional File 3). *Sa_v *dsRNA was diluted in injection buffer and injected into the dorsal heart vessel of freshly hatched first instar nymphs. In three independent injection experiments, stripes one and two, which are formed during the first and second nymphal instar, developed a persistent brick-red to light brown coloration in over 15% of injected animals (Table 1). This coloration contrasted with the dark brown to black color of the corresponding stripes in control animals and also of the posterior region of the eye, which is formed earlier during embryogenesis (compare panels 1a-c with f-h in Fig. 1). The phenotypically visible reduction of ommochrome formation in first and second eye stripes was indicative of reduced *Sa_v *gene activity following dsRNA injection in the first instar nymph. The persistence of ommochrome pigmentation in the posterior partition of the eye was consistent with the known long half life of ommochrome pigment granules [23].

Without exception, the third stripe formed in *Sa_v *phenotypic animals emerged in darker coloration than the stripes one and two, matching the coloration of eye stripes in non-treated animals at this stage of development (compare panels c and d with panels h and i in Fig. 1). The difference between stripe three and the previously formed stripes one and two suggested that *Sa_v *expression returned to normal levels by beginning of the third instar. This implied that the duration of *Sa_v *RNAi mediated gene knockdown was limited to the first two nymphal instars, which corresponds to 10 to 14 days under the culture conditions chosen. Stripes one and two remained distinct through the fifth nymphal instar (Fig. 1i). Lighter coloration and a narrower width of stripes one and two were still noticeable in adult animals suggesting that differentiated retina cells do not or just very slowly replenish ommochrome content (Fig. 1j).

No additional differences were detected in affected animals besides the transient change in eye coloration. Animals injected with dsRNA covering 300 basepairs of the Enhanced Green Fluorescent Protein-1 (EGFP-1) gene developed like non-injected animals and did not exhibit detectable morphological abnormalities (Table 1). In summary these results demonstrated that a single dsRNA surge in the grasshopper nymphal hemolymph elicits specific gene knockdown over a time course of 10–14 days.

### Quantitation of systemic RNAi mediated knockdown efficiency

**Figure 2:**
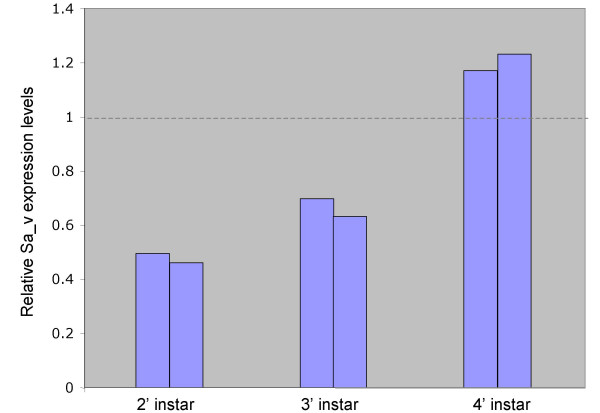
**qRT-PCR quantitation of systemic RNAi induced *Sa_v *gene knockdown. **Bars represent relative *Sa_v *expression levels of phenotypic grasshopper nymphs in the second, third, and fourth instar compared to expression levels in non-treated reference animals.

To confirm the *Sa_v *gene knockdown and measure its efficacy we investigated the relative levels of *v *expression in phenotypic animals by qRT-PCR (Fig. 2). These experiments revealed a two fold decrease of expression levels in second instar nymphs which rose to a 1.5 fold decrease in third instar phenotypic nymphs. Consistent with the re-initiation of normal pigmentation in the third stripe of fourth instar nymphs, *Sa_v *expression levels at this stage were not reduced compared to non-treated animals. Instead, slightly increased expression levels were observed which may reflect increased transcriptional activation of *Sa_v *in response to a high tryptophan titer in the transiently silenced animals. Overall, the course of *Sa_v *expression level changes in affected animals was consistent with the time window of eye coloration change.

## Discussion

The present study represents the first example of targeted and specific gene expression manipulation in grasshopper. It takes advantage of the evolutionary conservation of the systemic RNAi pathway in this species. To demonstrate and investigate efficacy and specificity of this gene silencing pathway we chose a widely used target gene, the *v *encoded tryptophan oxygenase, which catalyzes the first step in the biosynthesis of ommochrome eye colour pigments. Our results confirm the suitability of the ommochrome synthesis pathway for the evaluation of lack of function gene manipulation methods [24,25]. Even though qRT-PCR indicated a remaining 50% of normal expression levels in affected animals, a resulting change in eye coloration could be clearly and easily detected in life animals and monitored over time. The ommochrome biosynthesis pathway thus represents a sensitive and convenient real time readout of gene dose reduction. Evolutionary conservation, ease of interference and the normal viability of hypomorphic animals under laboratory conditions render *v *and the ommochrome pathway a widely applicable target to evaluate loss gene function methods in insects.

Our data also represent the first demonstration of systemic RNAi induction in the juvenile stage of a primitive insect. It has been previously reported that localized application of dsRNA into the cavity of the cercus appendage in cockroach nymphs silenced target gene expression in cercal sensory neurons but not in the more distantly located neurons of the ventral nervous system [26]. These observations implied the existence of dsRNA uptake mechanisms but indicated limitations in either the reach of injected dsRNA or the accessibility of different tissues. The present results in grasshopper nymphs demonstrate the induction of gene silencing at far distance from the entry point of dsRNA injection. Insect tryptophan oxygenase is expressed in many tissues including fat body, Malpighian tubules, gut and epidermis [23]. The qRT-PCR data specifically reveal a change of *Sa_v *expression levels in tissues of the head. The change of eye coloration, however, is most likely due to the non-autonomous effect of *v *activity reduction which leads to a drop of heamolymph levels of kynurenine, the starting substrate of the ommochrome synthesis pathway. Additional evidence for systemic RNAi competence of a wide range of grasshopper tissues comes from the observation of ubiquitous expression of *sid-1 *in embryonic and postembryonic tissues. Consistent with this, additional experiments in our laboratory targeting cell autonomous eye selector genes result in specific perturbations of compound eye formation (Dong&Friedrich, in preparation).

The penetrance of systemic RNAi induced *v *knockdown in grasshopper is much lower than that reported for honey bee (95%) and flour beetle (100%) [15,17]. Likewise, the temporal perdurance of the silencing effect seems shorter that in honey bee (25 days) and flour beetle (>4 months) [15,17]. It remains to be explored if these differences are caused by species specific differences in the systemic RNAi machinery or technical aspects. The larger body volume of grasshopper nymphs compared to the juvenile instars of honey bee and flour beetle is likely to lead to greater dilution of the injected dsRNA. Penetrance and perdurance of gene knockdown in the grasshopper may be increased by raising the amounts of injected dsRNA or by improving its delivery efficiency. The same strategies may also increase the efficiency of RNAi mediated knockdown which in the case of *Sa_v *reached only 50% reduction of transcription levels. The relationship between mRNA expression levels and RNAi induced phenotypes has not been quantitatively investigated in insects, which makes it difficult to compare our results with that in other systems [15,17]. However, it is reasonable to assume that approaching gene expression levels of heterozygous null mutants may not yield informative phenotypes with every gene examined. Nonetheless, additional data in our laboratory reveal that the expression of upstream regulators of grasshopper eye development can be reduced sufficiently enough to obtain informative phenotypes suggesting wider applicability (Dong&Friedrich, in preparation). Furthermore, the detection of *sid-1 *expression grasshopper ovaries raises the possibility that parental RNAi can be induced, which may reduce mRNA levels in the embryo more efficiently.

## Conclusion

Our data demonstrate conservation and expression of the dsRNA channel protein gene *sid-1 *in grasshopper, a primitive insect. Consistent with this, systemic application of dsRNA sequence identical to the grasshopper eye coloration gene *v *leads to specific, long-term and phenotypically visible reduction of endogenous grasshopper *v *gene activity demonstrating the conservation of the systemic RNAi pathway in grasshopper. In the light of these results we are confident that the conservation of the systemic RNAi pathway paves the way for the molecular dissection of development or neuronal processing in this primitive insect model system. The systemic RNAi pathway may also harbour potential for the development of species specific, and hence ecologically friendly pest control methods.

Based on the conservation of *sid-1 *in primitive and higher insects, it is reasonable to assume that the systemic RNAi pathway is conserved in a wide range of primitive insect species. Recent reports of parental RNAi induced gene knockdown in the milkweed bug *Oncopeltus fasciatus *and the two-spotted cricket *Gryllus bimaculatus *support this conclusion [27,28]. Nymphal RNAi, the systemic RNAi induced gene knockdown in juvenile stages of primitive insects, is therefore likely to develop into a valuable experimental tool analogous to the larval RNAi technique established for higher insects [17].

## Methods

### Animal culture

*Schistocerca americana *nymphs were obtained from an in house laboratory culture maintained as described [7].

### Molecular cloning

For RT-PCR cloning experiments, total RNA was isolated from first instar nymphal heads with the RNAqueous Kit (Ambion). cDNA template was prepared from total RNA with random decamer or Oligo(dT) primers using the RETROscript™ kit (Ambion). Two rounds of PCR amplification were carried out with nested pairs of degenerate primers targeting conserved gene regions determined by alignment of published *sid-1 *homologs from distantly related species (see Additional data file 1) (primer sequences available on request). PCR fragments were cloned into the pGEM-T vector system (Promega) and plasmid DNA purified with the Perfectprep Plasmid Mini kit (Eppendorf) for sequencing with the BigDye Terminator Sequencing kit (Applied BioSystems). Sequence reads were prepared on an Applied Biosystems ABI Prism 3700 sequencer in the Applied Genomics Technology Center of Wayne State University. Sequence analysis and alignments were carried out with MacVector 7.2 (Oxford Molecular Group).

### *Sid-1 *expression analysis

Whole mount *in situ *hybridization to examine the regulation of *sid-1 *expression in grasshopper embryos at 40% of development was carried out as described [7]. Life cycle and tissue specificity of *sid-1 *expression was determined by RT-PCR with specific primers (sequences available on request) on cDNA prepared from embryos at 40% of development, second instar nymphal head and abdomen, and adult eye, leg muscle and ovaries. RNA preparations were obtained by use of the RNeasy Mini kit (Qiagen) followed by DNA removal with Rnase-Free Dnase set (Quiagen).

### Grasshopper nymphal RNAi

dsRNA was prepared by bidirectional in vitro transcription from PCR templates following published protocols [17]. Freshly hatched first instar nymphs were immobilized by exposure to -20°C for 15 min. Injection solution containing 4 μl dsRNA at 5 μg/ul concentration, 0.5 μl 10 × injection buffer (5 mM KCl, 1 mM KPO4) and 0.5 μl Phenol Red was backfilled into a needle pulled from 10 ul glass capillary tubes (Idaho Technology). 0.05–0.1 μl of dsRNA solution was injected into the dorsal midline between either the third thoracic and the first abdominal segment or the first and second abdominal segment using Eppendorf microinjector 5242 and a Leica compound microscope. Injected individuals were kept in mass culture during the first instar. Phenotypic animals identified in the second instar were cultured individually until reaching adulthood.

### qRT-PCR

RNA was extracted from the head of single individuals as described above. Primer combinations against grasshopper genes *v *and *armadillo *(acc# AF408424), which was used as normalizer, were designed using the Primer3 software  (primer sequences available on request). Optimization and measurement reactions were carried out using the SYBR green kit (ABgene) and the Promega Mx3000PTM Real Time PCR system following manufacturers instructions. Data analysis was done with the Promega Mx3000PTM Real Time PCR system software package. For each time point investigated, two phenotypic individuals were compared to two non-treated animals. Each bar in Figure 2 represents the average difference of one experimental animal to two reference animals. Each animal was represented by three independent reactions.

## Authors' contributions

YD designed the experimental strategy, performed all experimental work and helped drafting the manuscript. MF conceived the study and completed the manuscript.

## Supplementary Material

Additional File 1 **Alignment of *sid-1 *orthologous genes**. *Ce_sid-1 *= *Caenorhabditis elegans sid-1 *(acc# NP_504372), *Hs_sid-1 *= human *sid-1 *gene (acc# NP_060169), *Am_sid-1 *= *Apis mellifera sid-1 *(acc# XP_395167), *Sa_sid-1 *= *Schistocerca americana sid-1 *(acc# AY879097). Amino acid residues identical in more than one species are boxed. Click here for file

Additional File 2 **RT-PCR analysis of *sid-1 *expression**. *sid-1 *expression analysis. Lane 1: 1 kb DNA ladder (Invitrogen). Lane 2: Total embryo at 40% of embryonic development. Lane 3: Second instar nymphal head. Lane 4: Second instar abdomen. Lane 5: Adult eye. Lane 6: Adult leg muscle. Lane 7: Adult ovary. Lane 8: Negative control reaction. Click here for file

Additional File 3 **Alignment of *vermilion *homologous genes**. *Dm_v *= *Drosophila melanogaster v *(acc# NP_511113), *Tc_v *= *Tribolium castaneum v *(acc# AAL15466), *Sa_v *= *Schistocerca americana v *(acc# AY879098). Amino acid residues identical in more than one species are boxed. Click here for file

## Figures and Tables

**Table 1:** Penetrance and specificity of systemic RNAi induced *Sa_v *gene knockdown

Target gene	Total number of injected nymphs	Percentage of surviving individuals	Percentage of animals showing phenotype
*Sa_v*	92	75.0%	15.9%
*Sa_v*	114	86.8%	16.2%
*Sa_v*	51	56.9%	20.7%
EGFP-1	80	71.3%	0%
