# Supplementary figures and images for: Nymphal RNAi: systemic RNAi mediated gene knockdown in juvenile grasshopper
Source: BMC Biotechnol. 2005 Oct 3;5:25. doi: 10.1186/1472-6750-5-25 (PMC1266053; doi:10.1186/1472-6750-5-25)

## Slide 1
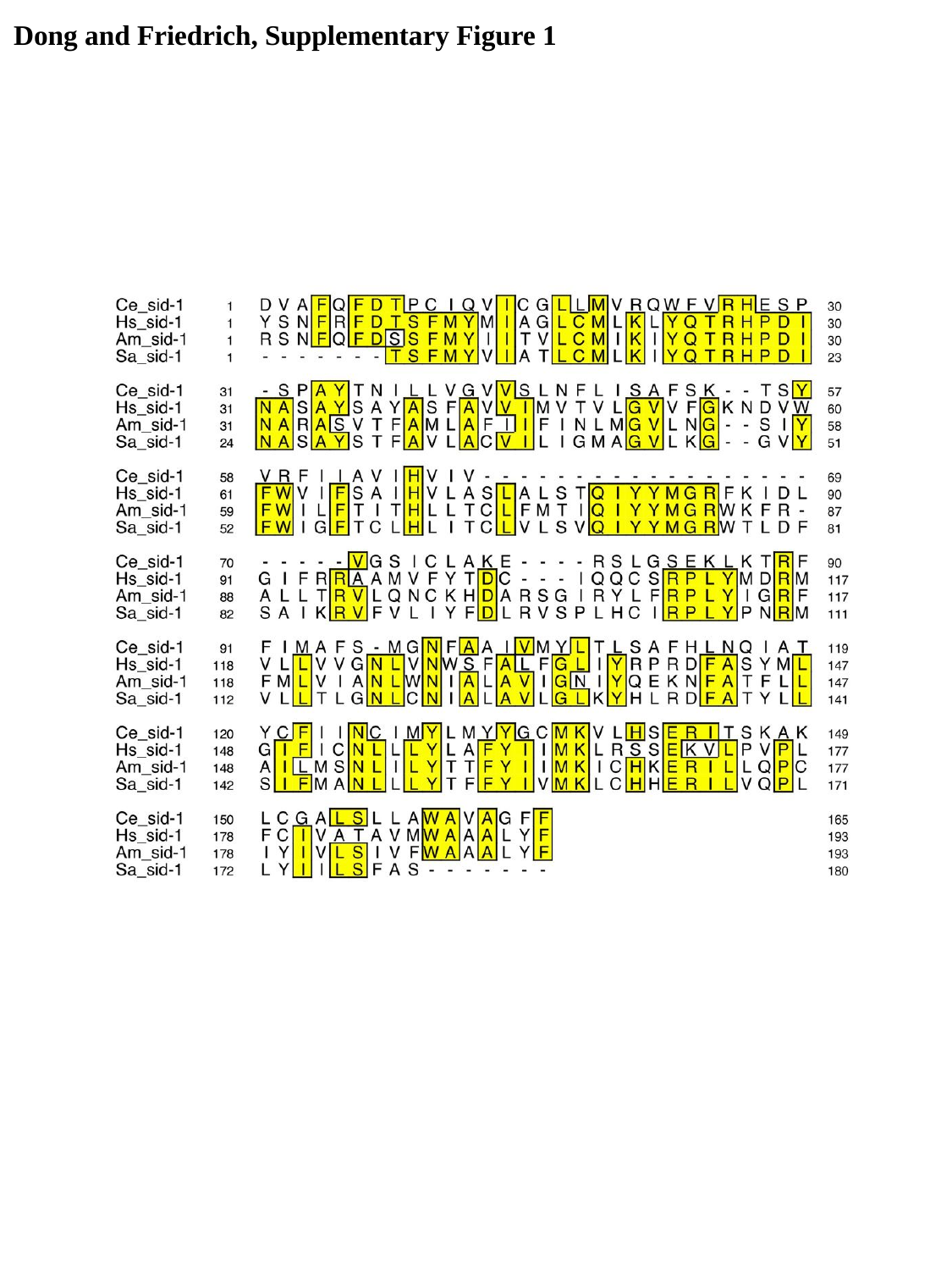

Dong and Friedrich, Supplementary Figure 1

Supplement: Additional File 1 — Alignment of sid-1 orthologous genes. Ce_sid-1 = Caenorhabditis elegans sid-1 (acc# NP_504372), Hs_sid-1 = human sid-1 gene (acc# NP_060169), Am_sid-1 = Apis mellifera sid-1 (acc# XP_395167), Sa_sid-1 = Schistocerca americana sid-1 (acc# AY879097). Amino acid residues identical in more than one species are boxed. [file 1472-6750-5-25-S1.ppt]

## Slide 1
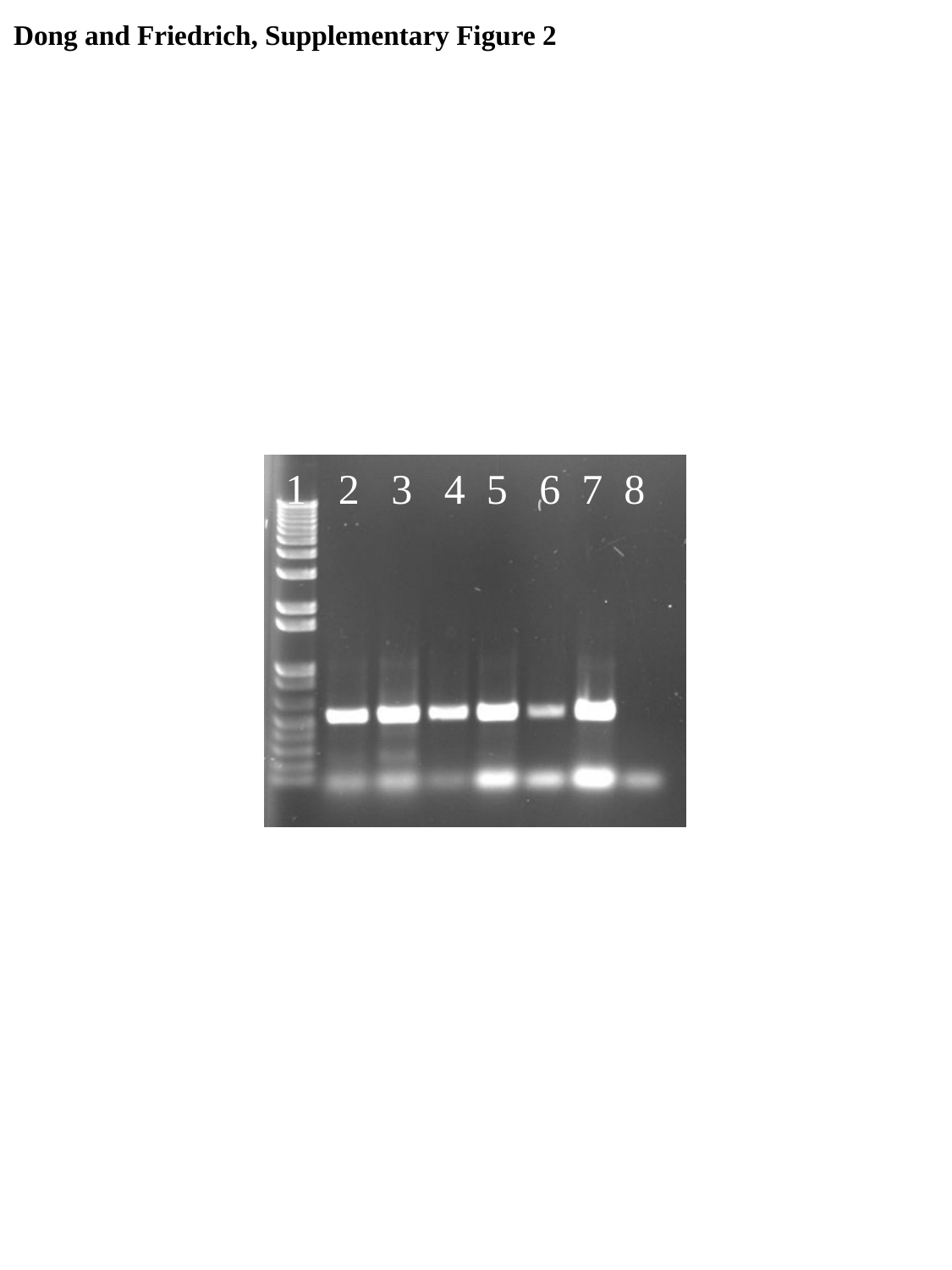

Dong and Friedrich, Supplementary Figure 2
1 2 3 4 5 6 7 8

Supplement: Additional File 2 — RT-PCR analysis of sid-1 expression. sid-1 expression analysis. Lane 1: 1 kb DNA ladder (Invitrogen). Lane 2: Total embryo at 40% of embryonic development. Lane 3: Second instar nymphal head. Lane 4: Second instar abdomen. Lane 5: Adult eye. Lane 6: Adult leg muscle. Lane 7: Adult ovary. Lane 8: Negative control reaction. [file 1472-6750-5-25-S2.ppt]

## Slide 1
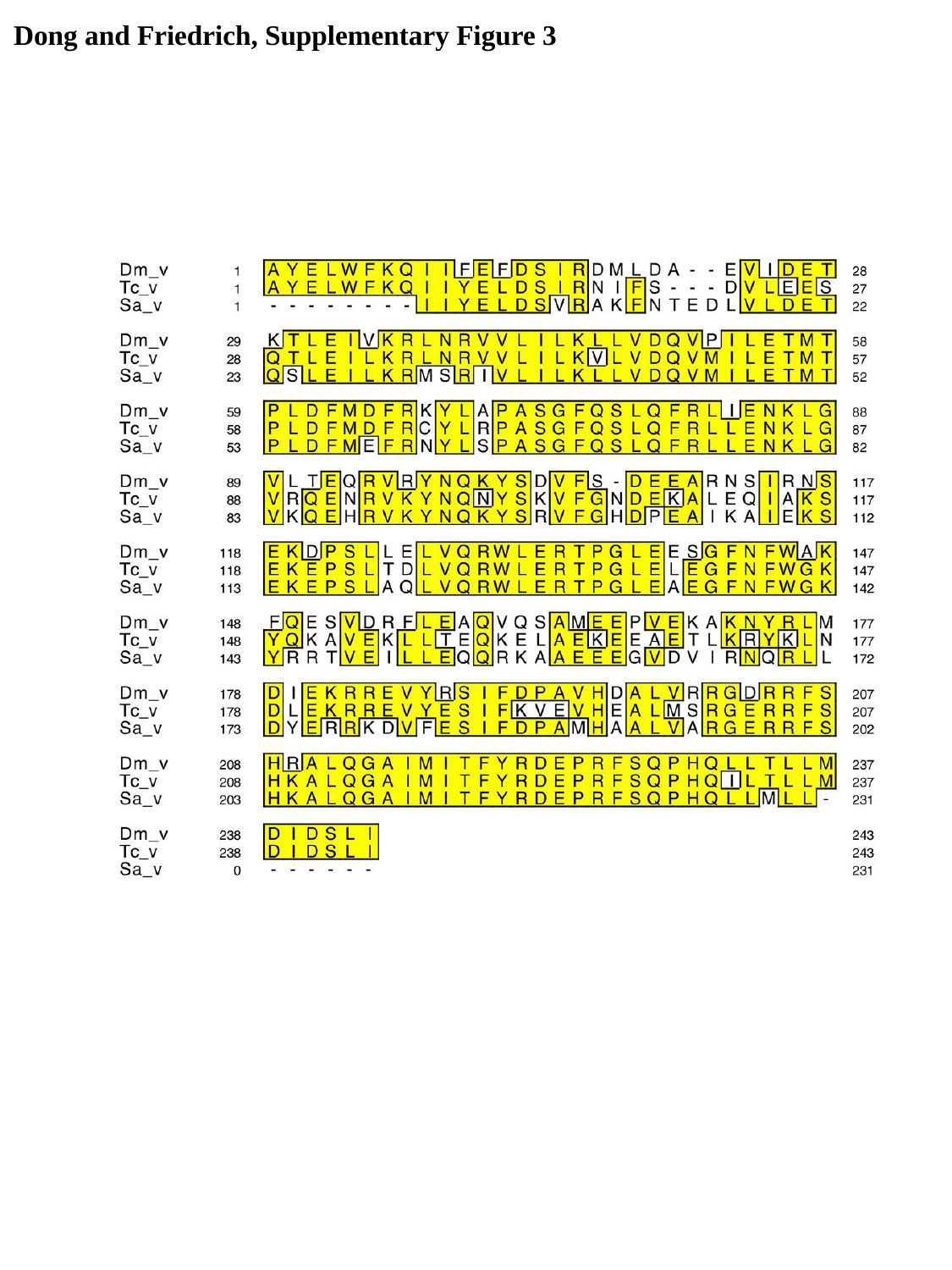

Dong and Friedrich, Supplementary Figure 3

Supplement: Additional File 3 — Alignment of vermilion homologous genes. Dm_v = Drosophila melanogaster v (acc# NP_511113), Tc_v = Tribolium castaneum v (acc# AAL15466), Sa_v = Schistocerca americana v (acc# AY879098). Amino acid residues identical in more than one species are boxed. [file 1472-6750-5-25-S3.ppt]
